# Developing laboratory capacity for Good Laboratory Practice certification: lessons from a Tanzanian insecticide testing facility

**DOI:** 10.12688/gatesopenres.13133.1

**Published:** 2020-06-12

**Authors:** Sara Begg, Alexandra Wright, Graham Small, Franklin Mosha, Matthew Kirby, Janneke Snetselaar, Salum Aziz, Jameel Bharmal, Russell Dacombe, Imelda Bates

**Affiliations:** 1 Liverpool School of Tropical Medicine, Liverpool, L3 5QA, UK; 2 London School of Hygiene & Tropical Medicine, London, WC1E 7HT, UK; 3 Innovative Vector Control Consortium (IVCC), Liverpool, L3 5QA, UK; 4 KCMUCo-PAMVERC Test Facility, Moshi, 255, Tanzania

**Keywords:** Laboratory, research capacity strengthening, good laboratory practice, insecticide, test facility, quality management system, quality management systems, capacity strengthening

## Abstract

**Background:** With increasing insecticide resistance in malaria-endemic countries there is an urgent need for safe and effective novel vector control products. To improve the capacity of facilities that test insecticides in sub-Saharan Africa, a programme is supporting seven facilities towards Good Laboratory Practice (GLP) certification, the globally recognized standard for quality management system (QMS) for the conduct of non-clinical and environmental studies. The World Health Organization (WHO) GLP Handbook provides guidance on a stepwise approach to implement a GLP compliant QMS. This study assesses auditor GLP checklists and timings outlined in the WHO GLP Handbook in the real-life context of a Tanzanian insecticide-testing facility, evaluating their implementation in this context.

**Methods and Principle Findings:** We conducted document review and semi-structured interviews with staff at all levels of the test facility to explore factors that influenced progress towards GLP certification. We found that while auditor GLP checklists underemphasised computer systems, they were otherwise broadly applicable. Factors that delayed time to completion of GLP certification included the need for extensive infrastructure improvements, the availability of regional expertise related to GLP, the capacity of national and regional external systems and services to meet GLP compliance requirements, and training development required for Standard Operating Procedure implementation.

**Conclusion:** The standards required for full GLP compliance are rigorous, with an expected completion timeline to implementation of 24 months. This study shows that in low and middle-income countries this timeline may be unrealistic due to challenges related to infrastructure development and lack of regional capacity and expertise. We recommend a comprehensive gap analysis when starting a project, including these areas which are beyond those recommended by the WHO GLP Handbook. These challenges can be successfully overcome and the experience in Tanzania provides key lessons for other facilities seeking GLP certification or the development of similar QMS.

## Introduction

The use of insecticidal mosquito control products is an important component of malaria control programmes in sub-Saharan Africa ^1, 2^. However, with insecticide resistance in malaria-endemic countries increasing, there is an urgent need to develop, test and commercialise new vector control products ^2, 3^. The World Health Organisation Prequalification Team for vector control products (WHO PQ-VCT) is moving towards a requirement for data for product evaluations to be generated only at Good Laboratory Practice (GLP) certified test facilities ^4^. The Organisation for Economic Co-operation and Development (OECD) Principles of Good Laboratory Practice set the quality standards for the organisation and management of test facilities and for performing and reporting studies. The OECD states:

*“the Principles of Good Laboratory Practice (GLP) are a managerial quality control system covering the organisational process and the conditions under which non-clinical health and environmental studies are planned, performed, monitored, recorded, reported and retained (or archived) …The Principles of GLP define the responsibilities of test facility management, study director, study personnel and quality assurance personnel that are operating within a GLP system, and minimum standards concerning the suitability of facilities and equipment to perform studies, the need for standard operating procedures, documentation of raw data, study reports, the archiving of records, etc.*” ^5^.

These principles are presented in the WHO Special Programme for Research and Training in Tropical Diseases GLP Handbook ^6^, supplemented by two training manuals, one for trainers ^7^ and the other for trainees ^8^. The purpose of a quality management system (QMS) compliant with the principles of GLP is to ensure that data generated during the conduct of non-clinical studies are reliable, repeatable and auditable. Data from GLP studies conducted in one OECD country must be accepted by other OECD countries and by non-OECD member countries adhering to the OECD System for Mutual Acceptance of Data for the purpose of assessment of chemical safety ^5^. This mutual acceptance of data, and the cost and time savings associated with it, is a key driver for speeding the registration and commercialization of new insecticides and hence justifies the need for more GLP certified laboratories with the capability of conducting laboratory and field studies on vector control products.

Laboratory capacity strengthening is an ongoing priority in low and middle-income countries (LMICs), most typically as part of a health systems strengthening agenda. Over the last decade, and initially driven by HIV and TB programmes, strategies have been developed to systematically strengthen clinical laboratories in LMICs ^9, 10^ with a view to improving the quality of data and the safety of laboratory personnel. These strategies have resulted in the development and widespread adoption of a process to support certification for clinical laboratories, the Stepwise Laboratory Improvement Process Towards Accreditation (SLIPTA) ^11^. SLIPTA is an auditing process against the international quality standard for clinical laboratories ISO 15189. This stepwise approach enables laboratories to map their progress towards meeting the ISO 15189 standard by assigning star ratings out of a possible five stars, according to their percentage of positive compliance against a defined checklist ^12^. ISO 15189 and SLIPTA are specific to clinical laboratories and are not suitable for use in non-clinical laboratories. Nevertheless, international quality standards are equally vital for non-clinical laboratories in LMICs, driven by the same factors as clinical laboratories; improved data quality and the safety of laboratory personnel. GLP is recognised worldwide as the quality gold standard for non-clinical laboratories.

The WHO GLP Handbook describes activities and personnel that are needed to successfully implement a laboratory QMS ^6, 13^. It recommends an initial ‘gap analysis’ conducted by a GLP expert based on an audit conducted over a four-to-five-day period. It describes through a step-wise approach how GLP certification can be achieved over a 24 month period, assuming that no GLP systems or documentation are initially in place ^6^. Unlike the SLIPTA process, the WHO GLP Handbook and stepwise approach have not been developed specifically for laboratories in LMICs.

IVCC ^14^, with funding from the Bill & Melinda Gates Foundation, supported this pilot study to review the work undertaken from 2014-2017 to achieve and sustain GLP at an insecticide-testing facility in Moshi, Tanzania. The test facility has been collaborating with IVCC since 2010 to develop a laboratory QMS. This is part of wider efforts to accelerate the speed with which new vector control products can be brought to market by strengthening research capacity at African test facilities. The facility is operated by the Pan-African Malaria Vector Research Consortium, which is an alliance of research institutions, laboratories and field sites in East and West Africa (Tanzania, Benin and Côte d’Ivoire) for the testing of new vector control tools and is based at the Kilimanjaro Christian Medical University College. In April 2017 it became the first facility in sub-Saharan Africa to be GLP certified. The certification process was undertaken by assessors from the South African National Accreditation System (SANAS) who conducted inspections using a ten-section checklist based on the OECD principles of GLP. As the SANAS checklist and the timing and activities for GLP certification described in the WHO GLP Handbook are based predominantly on experiences from non-clinical test laboratories in middle-high income countries, their relevance to and implementation in non-clinical laboratories in sub-Saharan Africa has not previously been evaluated. The purpose of the study, therefore, was to assess the applicability of the contents of the SANAS GLP checklist, to evaluate the feasibility of timings outlined in the WHO GLP Handbook in the real-life context of a Tanzanian insecticide-testing facility, and to learn lessons about how the efficiency of the GLP certification process could be optimised for other LMIC laboratories.

## Methods

### Study procedures

This was a mixed-methods case study. Activities undertaken by the test facility to achieve GLP certification and the time taken to complete each activity, were documented as they were undertaken. Records began in 2013 when the GLP process was initiated and were completed in 2017 when GLP certification was granted. These activities and their duration were compared with the contents of the checklist and to the recommended timings in the WHO Handbook, respectively, to identify activities that took longer than predicted. Semi-structured interviews were conducted with individual staff members involved in the GLP process to explore the underlying causes behind these divergences and to learn lessons about achieving GLP certification that could be applied to other African test facilities.

Ethical approval to conduct this research study was obtained from the Liverpool School of Tropical Medicine Research Ethics Committee (approval number 18-041). The study was included in the ethics approval for a wider IVCC study in Tanzania, obtained from the National Institute for Medical Research (approval number NIMR/HQ/R.8c/Vol.1/554). Participants were informed about the research using participant information sheets. Written consent was obtained from each participant prior to undertaking an interview.

### Document review and data extraction

The primary sources of data were the ‘to-do’ lists of the GLP Project Manager. These were created and retained to document the journey of the test facility towards GLP certification. These lists were supplemented with minutes from test facility meetings relating to the GLP certification process and facility audit reports produced by IVCC. Each activity undertaken as part of the GLP certification process was identified from these documents and listed, along with the date of the document, as an individual record. For activities that appeared on multiple documents, each date on which the activity was listed was recorded. The time in months between the first and last appearance of each activity was calculated, as was the total number of documents on which the activity appeared.

Each activity was mapped against requirements in the checklist using a pre-designed template based on the ten sections in this checklist ^15^.

1. Test facility, organisation and personnel
2. Quality assurance programme
3. Facilities
4. Apparatus materials and reagents
5. Test systems
6. Test and reference items
7. Standard operating procedures
8. Performance of the study
9. Reporting of study results
10. Storage and retention of records and materials

Each section is subdivided into the more detailed requirements for GLP compliance with a total of 149 requirements across all sections. The number of unique activities that had been undertaken for each section of the checklist was calculated, and activities that did not map directly to the checklist were listed separately and organised into groups for exploration through interviews.

The WHO GLP Handbook outlines 45 steps that must be completed in order to achieve GLP compliance, arranged in a set order and time (in months) that each step should take, with a minimum duration of two months and a maximum duration of six months. Each activity that took at least two months to complete was assigned to the relevant step in the WHO GLP Handbook. This cut-off was used because two months was less than the minimum time allocated to any given step in the handbook. To assess how long each activity took compared to the times indicated in the WHO GLP Handbook, the difference between the time that each activity took and the expected time for that activity was calculated.

### Interviews

Activities that took at least four months longer to complete than the time outlined in the WHO GLP Handbook were explored through semi-structured interviews to investigate the underlying causes. This cut-off was applied to ensure that the number of activities explored (41) was practicable. A maximum-variation purposive sampling strategy ^16^ was used to capture the views of individuals involved at all levels of the test facility who had exposure to the GLP certification process. Twenty members of facility staff were included in study, with the intention of achieving theoretical saturation. These individuals included multiple representatives from each level of the organisation to triangulate different data sources and, hence, determine the reliability of findings. The interview topic guide was based on previous studies of laboratory capacity strengthening ^17^, with additional questions derived from the findings of the document review. Questions from the topic guide were selected to match the roles and responsibilities of the interviewee. Interviews were audio-recorded and transcribed in full. Interviews were conducted face-to-face in a private office within the test facility. Interviews lasted for between 25 and 75 minutes. All interviews were conducted by two researchers, one of whom had a technical understanding of GLP requirements in insecticide testing facilities, and the other had systems evaluation experience; neither of them had been involved in the GLP certification process at the test facility.

### Data analysis

**Figure 1.**
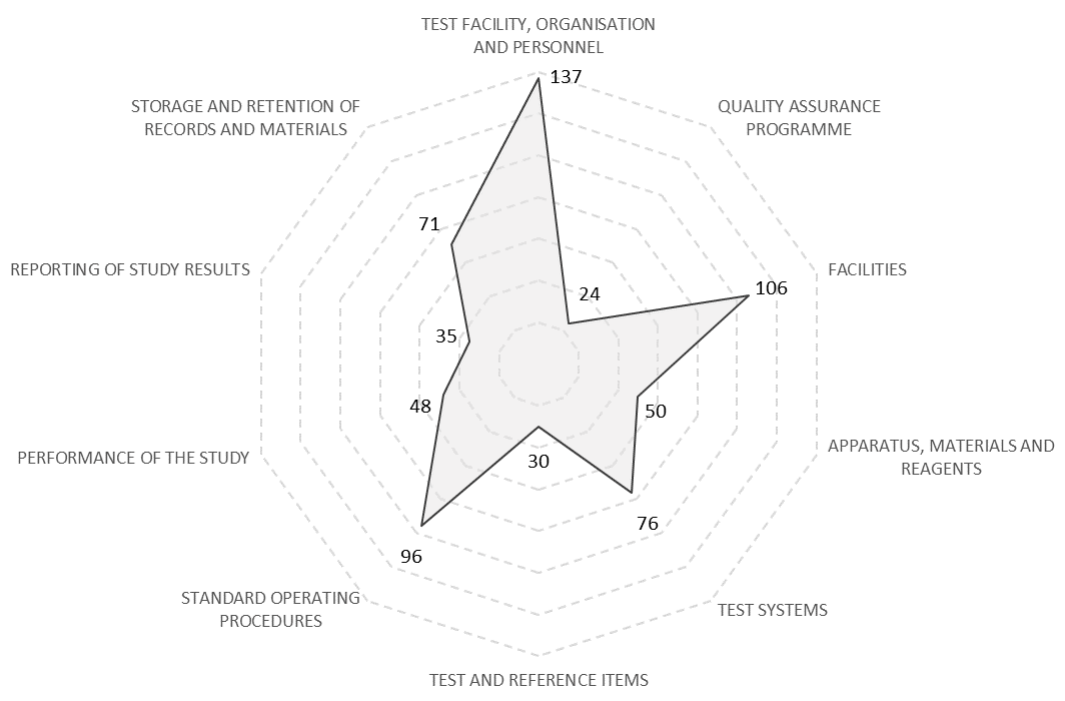
Number of GLP-related activities undertaken at the test facility allocated to the sections within the SANAS checklist. Unique GLP-related activities undertaken at the test facility were collated and allocated to the sections within the checklist used by the South African National Accreditation System checklist to audit GLP studies. Each axis in the radar chart represents a different section within the checklist, and the number of activities that were undertaken that relate to that section is charted. Very few activities were undertaken realted to the Quality Assurance programme, Test and Reference Items, and Reporting of Study Results. Many activities were undertaken related to the Test Facility Organisation and Personnel, Facilities, and Standard Operating Proceedures.

A framework analysis ^18^ was used to identify themes emerging from the interview transcripts following the five-step process of familiarization, identification of thematic framework, indexing, charting, and mapping/interpretation. The initial framework was based on the review of documents and therefore was structured around activities that had taken longer than the time suggested in the WHO GLP Handbook (Areas of Inquiry, Figure 1). Following familiarization with the interview data, further themes were identified and incorporated into the framework. All interview transcripts were indexed using NVivo 11 software (QSR International). To identify sections of the data that corresponded to the relevant theme, a narrative was constructed to define the key issues, to find associations between those issues, and where possible, provide explanations. Evidence from interview data was supplemented with relevant data from the document review to corroborate and contextualise this narrative.

## Results

A total of 28 documents were reviewed, spanning the period April 2013 to October 2016. These included 13 project management to-do lists or meeting minutes, eight internal audits, and seven audits conducted by IVCC. In total, 456 unique activities related to preparation for GLP certification were identified.

### SANAS checklist data

Each of the unique GLP-related activities undertaken at the test facility were allocated to the sections within the checklist where the activity contributed towards GLP compliance, with some activities contributing towards GLP compliance under more than one section ( *Underlying data*: Activities by SANAS Headings). The total number of activities that contributed towards each section was calculated ( Figure 1). Most of the recorded activities contributed to GLP compliance in the sections ‛Test Facility, Organisation and Personnel’, ‛Facilities’, and ‛Standard Operating Procedures’. Fewer activities were recorded that contributed to GLP compliance in the sections ‛Quality Assurance Programme’, ‛Reporting of Study Results’ and ‛Test and References Items’. This disparity was included as a subject for exploration during interviews.

A total of 48 activities (10%) did not correspond with any section of the checklist. These activities were related to basic laboratory decontamination, sanitisation and organisation (20), overall project management (17), HR processes (7), and staff welfare (4).

### Time taken to complete GLP activities compared to recommended time in WHO GLP Handbook

**Figure 2.**
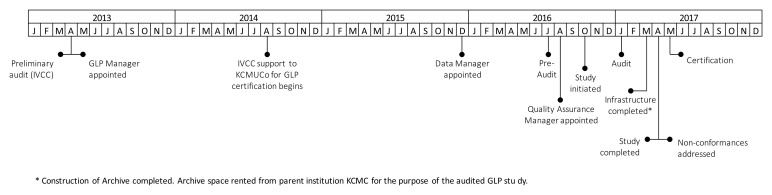
Timeline of GLP certification at the test facility. Key events related to GLP certification at the test facility between 2013 and 2017 were mapped on a timeline. The majority of key events were in late 2016/early 2017.

**Figure 3.**
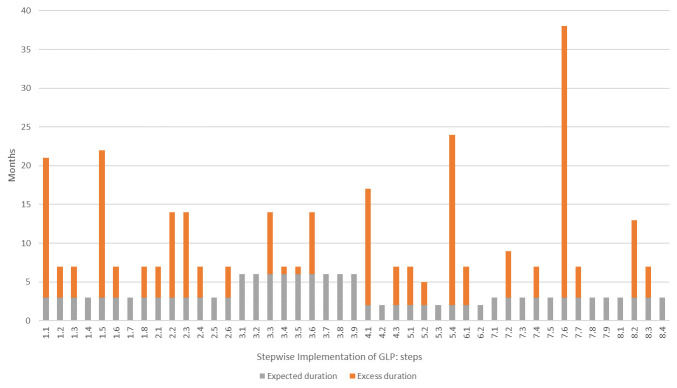
Additional time to complete activities required for each step in the WHO GLP Handbook. Each activity undertaken at the test facility was assigned to the step in the WHO GLP Handbook it corresponded to. The total time to complete each activity was calculated and compared to the expected duration according to the WHO GLP Handbook. The grey bars show the expected duration of each step, in months, while the orange bars show the excess time taken to complete the activities associated with that step. Five steps took over a year longer than expected. Step 5.4 (Define rules for the receipt, identification, handling, quarantine and husbandry of all test systems) and 7.6 (Formally train all staff in the use of the computer systems they need) took more than 24 months longer than expected.

From project instigation to final certification, the GLP project took 47 months, compared with 24 months suggested by the WHO GLP Handbook ( Figure 2). In total, 85 (19%) of the 456 GLP-related activities took at least two months to complete. Only 57 (67%) of these activities corresponded to steps in the WHO GLP Handbook. In addition, 27 (60%) of the WHO GLP Handbook steps had at least one activity that took longer than the expected duration to complete with a median excess duration of five months (range 1–35 months) ( Figure 3). Five steps included activities that took over a year longer than suggested in the handbook ( Table 1). All of the ‛delayed’ activities were grouped under the framework themes of training, data management and information technology, quality assurance, development and management of standard operating procedures (SOPs), and document control.

**Table 1.** Activities that took a year or more longer than suggested in the WHO GLP Handbook. Excess duration includes extended periods of inactivity, e.g. between identifying/booking training and training taking place and does not represent continuous work.

Step	Activity	Expected	Actual
1.1	Arrange general GLP training for all staff	3 months	21 months
1.5	Compile the personnel documents for all staff using the formats agreed upon in 1.4 above	3 months	22 months
4.1	Establish a Quality Assurance Unit	2 months	17 months
5.4	Define rules for the receipt, identification, handling, quarantine and husbandry of all test systems (i.e. insectary facilities)	2 months	24 months
7.6	Formally train all staff in the use of the computer systems they need	3 months	38 months

The WHO GLP Handbook states that establishment of the Quality Assurance unit (steps 4.1 to 4.3) should take two months and should begin 12 months into the project; at the test facility this took 17 months. Computer systems and data management are first addressed in steps 7.1 to 7.9 of the handbook, 18 months into the stepwise approach, and are listed as requiring 3 months to complete; at the test facility this took 38 months.

A total of 27 activities took at least 2 months to complete and were not included in any of the WHO GLP Handbook steps. Of these, 12 activities took more than a year to complete. These activities were related to health and safety (including personal protection equipment) and implementing systems to prevent contamination particularly of resistant and non-resistant mosquito strains ( Table 2). Seven of the remaining activities were related to construction and restoration of facilities for GLP compliance purposes.

**Table 2.** Activities requiring more than 12 months to complete and were not included in WHO GLP Handbook steps.

Activities	Duration (months)
Laboratory coat rack - test facility laboratory coats only	26
Shoe racks and plastic containers for dirty and clean shoes, shoes labelled (resistance and non-resistance sides)	26
Improve toilets - soap and water, paper towels, lighting	26
Lockers for staff, with locks and personal items locked away	25
SOP for confidentiality	21
SOP for visitors to facilities and field sites	21
Safety manual file, signed and dated	14
Fire extinguisher - form daily, archived monthly, training with Fire Inspector, drills and assembly point	14
Eye wash form next to eyewash - daily	13
General tidiness	13
Trash can with plastic bags in each room	13
Install whiteboard	12

SOP – Standard Operating Procedure

### Exploration of reasons for delayed activities

Twenty staff members were approached for interview and none declined to take part in this study. Of these staff, 5 were laboratory/insectary technicians or attendants, 4 were from non-scientific administration/information technology positions, 7 were from scientific middle-management positions, and 4 were from scientific senior management positions. Due to the small number in each staff cadre, anonymised identifiers have not been used for quotes from transcripts. From the interviews, four overarching factors were identified as significant influencers on the rate at which the test facility progressed towards GLP certification. These were: the timing, content, and providers of training on the principles of GLP for the test facility staff; the recruitment to key roles of individuals with relevant expertise or competence to develop the relevant expertise; the facility’s approach to SOP development and implementation; and touch points of the GLP QMS with external systems, agencies and organisations.

***Training in the principles of GLP*.** The WHO GLP Handbook recommends undertaking general GLP training with all staff as the first step in implementing the GLP system (Step 1.1, p63) ^6^ and states that “training of 1–2 days underlines the fundamental points of GLP and the importance of GLP for the organisation. Emphasis is placed on the way in which data are collected and handled”. Evidence from the document review indicated that, at the test facility, this task took 21 months to complete, because of delays in identifying an appropriate training provider and in setting up and implementing the training.

As the first insecticide testing test facility in Africa to work towards GLP certification, interpreting the GLP principles as they applied both to the science and to the context of the insecticide-testing facility was challenging. Initial training was provided in a series of sessions by collaborators from IVCC and a local Quality Manager. However, this did not fully meet the needs of staff, particularly because it was too generic, so additional training was provided by a specialised training provider connected to SANAS. This training outlined requirements for GLP certification from start to finish, clarified the roles that all staff played in achieving and maintaining GLP certification, and helped them adapt and apply the GLP principles to the test facility’s scientific field and context (e.g. how to set the acceptable ranges for environmental conditions in an insectary, given the limitations of the infrastructure).

*“The training with SANAS was really good, and it was specific to us. Whereas we had another training …. but it was not relating to us. Just because them they are much clinically, and they’re doing it with samples for patient blood samples.”*

Interview data indicated that gaps in the training remained, particularly in computer-based data management for GLP compliance. At the time of the study, computerised systems relating to GLP were mentioned in the overarching OECD guidelines, but practical details of how these should be implemented were lacking. Since the checklist and the internal and IVCC audits were derived from these guidelines, knowledge and implementation of computer-system based data management was under-represented in the GLP process.

***Recruitment of key roles*.** From the interviews it was apparent there was initially a general lack of staff understanding and engagement in the GLP process. This primarily related to ineffective communication about the significant changes and new roles and responsibilities involved in achieving GLP certification and was compounded by the lack of initial training in GLP. While all roles at a test facility pursuing GLP certification may need to adjust their working practices to meet GLP requirements, the roles of Quality Assurance (QA) Manager, Data Manager and GLP Study Director are absolutely key. Appointment of staff to these roles is listed in the WHO GLP Handbook as a task to be undertaken in the first three months, but in practice it took the test facility 15 months to accomplish. Data from the staff interviews indicated that this was due to a shortage of suitable applicants with relevant expertise who could be appointed directly to the post. Once appointed, the individuals needed extensive training and on-the-job learning before they were able to implement the systems and processes required by GLP. As an interim measure, an external international QA Manager was appointed which enabled the test facility to proceed with gap analysis and internal auditing. An internal appointment was made to the QA Manager post which overlapped with the QA Consultant, which gave the appointee time to attend international QA training in the UK and to develop QA tools and processes for the test facility.

Prior to implementing GLP standards, the IT department at the test facility consisted of one part-time member of staff. Interviews revealed that there was also a general lack of awareness of, and expertise in, the validation of computerised systems for GLP compliance. This lack of awareness was reflected in early internal audits which did not highlight the gap in computer systems validation as a major non-conformance in the facility. These factors together led to major delays in the development of the Data Management system for GLP. Once the GLP project team became fully aware of the importance of filling the gap in computer systems validation, a full-time IT and Data Manager was appointed. However, it took 38 months before recruitment and training was completed and systems were developed at the test facility, compared to the 3 months suggested in the WHO GLP Handbook.

*“The whole process was a bit of a problem because like at least in other departments, it's a bit easy because the study director or someone who could actually know how to implement certain things but in the computerized system, it was like everyone was a layman.”*

The initial lack of awareness in the GLP certification process also affected the role of senior staff at the test facility, leading to a four-month delay in the production and approval of some QMS documentation. The situation at the test facility was particularly complicated because the nominated study director’s job description did not initially include working on the GLP process, which placed an additional burden on their workload. The situation was eventually alleviated by employing a full-time GLP Manager to support the Study Director and to act as a bridge between technical and managerial staff.

***Standard Operating Procedure development and implementation*.** SOP development and implementation required both a substantial amount of time and human resource at the test facility, as highlighted by the number of activities related to SOPs identified in the document review (96/456, 21%). The development of SOPs (and associated training to ensure that all staff were competent to follow and not deviate from SOPs, including accurate completion of documentation) placed a substantial burden on staff in supervisory and Study Director roles. The number of SOPs developed and implemented at the test facility increased from 23 in 2014 to 120 by the time of the final audit in 2017. Since this was the first test facility in sub-Saharan Africa to seek GLP certification, many SOPs had to be written from scratch or, if adapting from guidance from WHO or similar organisations, had to undergo substantial revisions to address contextual challenges.

The UK Research Quality Association provided training courses, junior management, first aid and fire training, and ‘Introduction to GLP’: all other training was generated in-house. Training pathways, assessments and criteria to demonstrate competence/expertise were developed in-house for almost all practices and procedures. The development of the whole training programme required a considerable investment of time and was necessitated by the lack of viable alternatives in-country as the test facility was the sole expert in-country on the techniques used. From the interviews with staff members, it was clear that the collaborative approach to SOP development and training was regarded as being effective but time consuming. SOPs were developed by documenting existing best practice, with technicians completing procedures whilst managers documented them, with further elements required for GLP compliance also being incorporated. The training process for SOP implementation was a highly iterative process with supervisors reviewing SOP deviations and identifying and resolving the root causes. This process was well-regarded by staff and they recognised that involvement of staff at all levels in the SOP development process enhanced motivation and engagement with the wider GLP project.

*“The process is good. It's good because it allows a person to have the theory of what he's expected to do so when he goes to do it, he already knows what he's supposed to do. If he sees difficult, it is easy to mention that, ‛The SOPs say this way, but I find it difficult to work at this way.’ It's another way we can improve the SOPs or improve training for that person. I think it's a good process, going on well and the guys like the process.”*

***Touchpoints with external systems, agencies and organisations*.** To achieve GLP certification, the test facility and GLP project management team needed to interact with external systems, agencies and organisations ( Table 3). Interviews indicated that these interactions presented several challenges resulting in delays to progress on the GLP project.

**Table 3.** External systems, agencies and organisations' interactions with the test facility GLP project.

	External system interacting with GLP activity				
	Waste Management	Animal Husbandry	Calibration	Importing	Construction and Land Ownership
*GLP* *requirement*	Meeting national guidelines.	Meeting national guidelines.	Must be conducted by an officially accredited calibration laboratory.	Some equipment and consumables required for creating a GLP-compliant environment and to run a GLP study are not available to purchase in Tanzania.	Infrastructure must be adequate for the completion of the study, including enough space per person in a test facility and adequate separation within the test facility to prevent contamination.
*Challenge*	Required test facility visit from National Environmental Management Council.	Inadequate national guidelines exist for animals for feeding mosquitoes, (and study funders may have additional requirements).	No calibration laboratories in Tanzania with the required accreditations. Inadequate shipping arrangements to nearest laboratory in Kenya undermined calibration.	Long shipping times, changing systems of import permits, and lack of clarity as to which government body should provide permits for importing some equipment and consumables.	Land at the test facility was owned by a separate trust and permission had to be obtained to build new structures or refurbish existing structures.
*Outcome*	Arranging the test facility visit took a year and was followed by several further months identifying which processes (e.g. incineration, charcoal- based deactivation of pesticides) should be followed.	Examples of best practice researched and modified for context, which were adequate for funders. Contributions made to future national guidelines.	Calibration provider identified in South Africa, with appropriate shipping requirements. Budget for calibration increased.	Significant delays in importing, resulting particularly in delays to ensuring environment was GLP compliant. Implementation of rigorous stock management system and procurement administration processes.	Significant delays to beginning construction, as well as substantial time burden for senior members of staff at the test facility. Some modifications were subsequently required with both cost and timeline implications.

GLP compliant waste management systems and animal husbandry (for the test facility this needed to be adapted to be relevant for the testing of mosquito vector control products) must align to national guidelines and regulations:

“It should also be assured that unused test and reference items are returned to the sponsors or suppliers or **are disposed of in a legal and responsible manner.**” (WHO GLP Handbook, p185) ^6^

“Housing conditions and the way animals are treated must satisfy the scientific needs of the study **and accommodate national animal welfare legislation.**” (WHO GLP Handbook, p31) ^6^

If these national guidelines do not exist or are not adequately detailed, as was the case in Tanzania, appropriate alternative guidelines need to be adopted for GLP compliance. Interview data indicated that for waste management, the Test Facility Managers spent more than a year organising a test facility visit with the relevant regulatory authority in order to develop a plan that would be appropriate and acceptable. In the case of animal husbandry, the Veterinary Council of Tanzania and the District Veterinary Office were engaged, a facility audit was conducted, and the test facility was registered as a breeder of animals for research purposes, demonstrating compliance with Animal Diseases Act 2003 and Animal Welfare Act 2008. However, the guidelines underpinning these Acts were generic and did not provide the detail necessary for implementation in the specific context. The Test Facility Managers therefore modified international examples of best practice to suit their context, taking into consideration the animal welfare requirements of ethics committees of partner institutions and funders. For both waste management and animal welfare there were communication delays with national authorities resulting in additional time required to complete these GLP components.

Engagement of accredited service providers and suppliers outside of Tanzania was necessary for both maintenance and calibration of instruments, and for procurement of many supplies as contractors that met the requirements for the GLP compliance of the test facility were not available in Tanzania. Defining and implementing processes for maintenance, metrology and qualification of instruments is predicted to take 6 months in the WHO GLP Handbook but took 25 months at the test facility after a SANAS-accredited South African service provider was identified. Using a service provider accredited by the same authority as that which was auditing the test facility was useful for inspectors and the Test Facility Managers, but this led to additional costs including that for the annual service provider visits. In addition, because the calibration turnaround time using international contractors can be six to nine months, a second set of instruments had to be purchased so that instruments were always available for use in studies. This required careful planning of study timelines to ensure instruments were always available when required.

International procurement of some equipment (e.g. personal protective equipment, fire-resistant cabinets) was necessary as accredited suppliers of equipment of acceptable quality were not available in Tanzania. This added to both time and costs and required good stock management to ensure that materials were available for GLP studies. Import permit requirement for products, including for vector control products, were inconsistent and necessitated interactions with many agencies including the government chemist, the Tropical Pesticides Research Institute and the Tanzania Bureau of Standards, which further added to delays.

Interviewees highlighted that, at the test facility, substantial and time-consuming changes to the test facility infrastructure (including the construction of new buildings and refurbishment of existing buildings) were required to meet GLP compliance requirements, and that these changes were not included in the WHO GLP Handbook. Changes to facility infrastructure required negotiations between the Test Facility Managers and the landowners, and also close supervision of the construction teams to make sure the buildings were of the required quality. The infrastructure changes incurred substantial financial and time costs. This had the knock-on effect of delays in the final training and testing of SOPs, as these could only be completed once changes to infrastructure had been completed.

## Discussion

The purpose of the study was to assess the applicability of the contents of the SANAS GLP checklist, to evaluate the feasibility of timings outlined in the WHO GLP Handbook in the real-life context of a Tanzanian vector control product test facility, and to learn lessons about how the GLP certification process could be optimised for test facilities in other LMICs.

### SANAS GLP checklist

The SANAS GLP checklist is used for auditing purposes during the inspection of test facilities that wish to be granted or to maintain GLP certification. The checklist is derived from the OECD GLP document No. 1 ‘OECD Principles on Good Laboratory Practice’ ^19^ and provides a good framework for the inspection of test facilities for GLP compliance. Therefore, this study found that the vast majority of activities undertaken at the test facility corresponded to requirements described (438 activities out of a total of 486) in the checklist. However, as the checklist is derived from the overarching OECD Principles on Good Laboratory Practice, it does not include all of the information relating to guidance on the application of the principles of GLP to computerised systems which, at the time of this study, were outlined in OECD GLP consensus document No. 10 ^20^. Furthermore, as OECD GLP consensus document No. 10 was developed in 1995, it did not accurately reflect recent changes in the use of computer systems at test facilities. Consequently, this area was under-scrutinised by the Project Management team. In the time the test facility was granted GLP certification, a new advisory document (OECD GLP document No. 17 ‘Application of GLP Principles to Computerised Systems) has been issued which replaces OECD GLP document No. 10, and outlines the application of GLP principles to computerised systems to reflect current technology ^21^. Rapidly changing technology has previously been highlighted as a challenge when improving QMSs in Tanzania ^22^. Aside from the lack of emphasis on the development and validation of computerised systems for GLP compliance, the SANAS GLP checklist does not cover basic test facility organisation, decontamination and sanitisation which made up the remainder of activities undertaken.

### WHO GLP Handbook timelines

The WHO GLP Handbook outlines a stepwise approach to achieving GLP certification over a 24-month timeline. The timings outlined were sufficient for the completion of some steps as implemented at the test facility, in particular, the listing and management of equipment (apart from equipment calibration), and the preparation of some documentation related to the test facility organisation. However, the suggested 24-month timeline to certification was insufficient for achieving full GLP compliance in the real-life context of the Tanzanian test facility. In particular, the Handbook underestimated timings related to infrastructure development, knowledge of and training in GLP, appointment and training of key roles, and interactions with national/regional supporting infrastructure. Interview data further revealed that SOP development and implementation was a particularly time-consuming process that resulted in delays to progress.

### Infrastructure development

Lack of good quality infrastructure is a recognised barrier to laboratory capacity strengthening efforts in LMICs ^22, 23^. While the OECD guidelines for GLP include requirements for laboratories to meet infrastructural standards – for example, the minimum amount of space per member of staff working in a laboratory and adequate separation between sections of the laboratory – steps related to infrastructural improvements, including building construction, tiling of floors and walls, and installing appropriate benches and storage units, are not included in the WHO GLP Handbook stepwise approach and associated timelines; at the test facility this required substantial time and effort. The WHO GLP Handbook stepwise approach assumes that infrastructure at test facilities seeking GLP certification is already fit for purpose and, in terms of physical resources, focuses in the most part on the equipment within the facility. Although absent from the WHO GLP Handbook stepwise approach, construction and infrastructural changes were time consuming at the test facility and required negotiation of permissions, international procurement, and close management to ensure the project was delivered to the specifications required for GLP compliance. As the OECD GLP guidance documents include requirements related to facilities infrastructure, this should be reflected in the WHO GLP Handbook’s stepwise approach, with an indication of the time that this may require and noting that this can run in parallel with some other steps such as non-laboratory specific documentation and recruitment (see below).

### Knowledge of and training in GLP

GLP is a QMS intended to assure the quality and integrity of non-clinical laboratory studies generating data for regulatory purposes. Knowledge of QMSs is frequently cited as a barrier to progress in laboratory capacity strengthening projects in LMICs ^22– 26^, particularly a lack of knowledge of staff within the laboratory ^25^ and a reliance on external consultants to support the development of quality systems ^26^. In the absence of internal GLP expertise, the test facility needed to identify a provider of appropriate general GLP training within the region. This proved challenging and a piece-meal approach to delivering this training was necessary in the early stages of the project, calling on expertise within the project team and within the region. This issue was ultimately resolved by contracting a specialised agency who had in-depth knowledge of the site through connection to the accrediting body SANAS, to deliver general GLP training following the first inspection.

The progress of the test facility may have been accelerated had SANAS’s bespoke GLP training been delivered at the start of the project, given the strong preference for this training expressed by the test facility staff. This, however, assumes that GLP training would have been as effective prior to the SANAS inspection by the certifying body as it was post-inspection. Post-inspection, GLP training delivered by SANAS could be tailored to the types of studies conducted at the test facility as the training was delivered after the first inspection. In reality, this would not be a feasible option for all test facilities implementing a GLP QMS as, in regions where the GLP monitoring authority may not have been exposed to the type of studies being inspected, they would not be able to deliver bespoke training. Therefore, sites may wish to consider planning for training between pre-inspection and the full inspection to allow inspectors/trainers to gain a good understanding of the facility and be able to develop and deliver a bespoke training programme prior to the full audit.

### Appointment and training of key roles

Whilst all staff at a test facility seeking GLP certification must comply with the requirements described in the OECD principles of GLP, some roles were found to be of particular importance at the test facility, namely Study Directors, the IT and Data Manager and the QA Manager. The test facility had appropriately trained and experienced Study Directors in post prior to undertaking the GLP project, but this was not the case for the IT and Data Manager and the QA Manager roles. Both roles were new or expanded positions at the test facility and identifying appropriately trained and experienced candidates from within Tanzania proved to be challenging. Ultimately, insufficient training of the IT and Data Manager was highlighted as a non-conformance by SANAS which had to be addressed post-inspection. This is consistent with findings from the wider laboratory capacity strengthening literature ^22– 25, 27^ where insufficiently trained and inexperienced staff are commonly cited as barriers to progress. At the test facility, this was addressed through a variety of approaches, including the temporary use of external consultants, internal promotion of staff and the appointment of new staff. In addition, the test facility invested in expanding the individual level capacity of staff appointed to the Study Directors, IT and Data Manager, and QA Manager through external QA and data management training by the UK RQA and on-the-job support. Individual characteristics of staff appointed to these roles may have been a factor in the success of this approach, as the individuals were prepared to undertake significant independent study as well as planning and problem solving abilities, to understand, interpret and apply the GLP requirements as applicable to the context of studies conducted at the test facility.

### National/Regional availability of supporting infrastructure

This study has highlighted the importance of access to relevant resources and supporting infrastructure which has previously been identified in the context of health laboratory capacity strengthening ^22^, especially relating to procurement and importing of consumables ^22– 24, 28, 29^. This case study identified that, to achieve GLP certification, the facility required access to services which were either not available at all or were not available to the standard required for GLP compliance in-country. In the case of this test facility, these services related to waste management, animal welfare, accredited calibration laboratories, and sourcing importation permits from the correct regulatory bodies. GLP guidelines indicate that, if essential services are not available, facilities may, in the short term, develop their own bespoke approaches (as was the case for waste management, animal welfare and in-house training in SOPs) or use alternative service providers (as was the case for calibration services). However, these alternative approaches added to the cost of obtaining GLP certification.

### SOP development and implementation

SOP development is an ongoing process reflected in multiple stages in the WHO GLP Handbook. The task of developing and implementing SOPs was time intensive as the test facility started from a baseline of very few SOPs. The development of SOPs led to significant delays to progress, particularly as it was the first insecticide testing facility in sub-Saharan Africa to seek GLP certification. Consequently, the opportunities for learning from best practices at other test facilities was limited. Nevertheless, there was a belief that the consultative, recursive approach to SOP development and implementation, although time-consuming, resulted in accurate and readable SOPs and in good levels of staff engagement. This corroborates evidence from the Democratic Republic of the Congo, where researchers found that the process of SOP development for a QMS was time consuming but that local staff input was vital ^30^. The development of the broader SOP training programme, including the development of criteria for assessments, was necessitated by the lack of viable alternatives. The test facility’s position as a GLP pioneer in insecticide testing facilities in Tanzania and sub-Saharan Africa means, therefore, that they are the only experts in-country on the techniques used. Overall this was a time-consuming task that put a substantial burden on senior staff members at the test facility.

### Limitations

This study is subject to several limitations. The first is the completeness of the document review in the initial phases of the project. Whilst documentation was collated as the work was undertaken, due to the nature of the project and particularly some of the major sources of delay in the project, some time periods had more documentation than others. This may have resulted in over or under estimation of the time to complete some activities. Best practice for similar studies would be to include a regular schedule for reviewing and updating to-do lists. The second limitation is that the interview component of this case-study was undertaken retrospectively, six months after the granting of GLP certification. As a result, the interviewees may not have been able to recall accurately all of the events during the previous four years of the GLP project. This was mitigated in part through the triangulation of data against real-time written records used in the document review. The final limitation is that this case study is focussed on a single test facility in Tanzania. As a result, the generalisability of these findings may be limited, influenced both by the national context of Tanzania and by the test facility’s position as a private, rather than a government, test facility. Future studies should include more test facilities from several countries, both public and private facilities, to identify which factors are context-specific and which hold true across most or all contexts.

### Lessons learnt

This descriptive case study outlines the factors that resulted in delays in implementing the GLP system at the test facility. Nevertheless, the test facility was able to overcome these challenges and successfully implemented a GLP compliant QMS and achieved GLP certification in April 2017. Whilst some of the challenges identified here may be test facility specific, with a further six facilities being supported by IVCC and a number of other laboratories by the WHO, future work related to this study will explore both the barriers and enablers to progress on GLP at vector control product testing facilities across both East and West Africa. There are some lessons that can be learnt from this case-study about how the effectiveness of the GLP certification process could be optimised for other LMIC laboratories.

Beyond allowing extra time to complete a GLP certification project, the overarching principle underlying the lesson learnt from this project is to conduct a comprehensive gap analysis at the start of the project and include additional areas beyond those recommended by the WHO GLP Handbook (summarised in Table 4). This will help test facilities identify potential risks that might impede progress towards GLP compliance, provide project teams with a clearer picture of the work they will have to complete, and allow for appropriate planning of project timelines.

**Table 4.** Key areas for inclusion in gap analysis at instigation of laboratory capacity strengthening initiatives towards GLP.

1. The infrastructural readiness of the site to deliver GLP studies: • Compliance with GLP requirements • Compliance with the requirements of study funders or collaborators (especially animal welfare) *Infrastructure rehabilitation must be completed before training in SOPs can commence, this must be addressed first. Some* *documentation can run in parallel to infrastructural improvements.* 2. The capacity of services outside of the laboratory to meet GLP requirements, including: • National policies on waste disposal • National policies on animal welfare • Regional availability of government certified calibration laboratories *Where these do not exist or are not able to meet GLP requirements, begin to find alternative solutions at an early stage in the* *project.* 3. The regional availability of individuals with experience directly related to implementation of GLP, in particular: • Training providers for general training in GLP • Quality Assurance for GLP • Data Management for GLP *If not already in position, Quality Assurance and Data Management roles should be appointed at the start of the project and* *training and capacity building should be an early priority*.

Project teams should identify what infrastructure improvements are required and initiate these improvements at an early stage in the project, with a plan to run these alongside the early documentation development phases of the project. Construction is likely to be time consuming and will require close supervision and project management, particularly when using non-expert construction teams; allowing for this additional time and work when developing project plans is strongly advised. When considering the capacity of external service providers to support GLP projects, we recommend that the project team make contact with relevant government agencies at the onset of the project, as a substantial cause of delay at the test facility was in identifying key individuals who could advise on appropriate solutions. In Tanzania, this was particularly important for waste management. For studies that require an animal test system or include animals in other ways (for example, rearing mosquitoes), we recommend that laboratories consider not only national guidelines on welfare but also the guidelines which collaborators and funders require the test facility to adhere to, which in some cases may be stricter. Data Managers and QA Managers should be an early priority for recruitment and/or training. If it is necessary to use training providers outside of the region, the logistics and travel expenses associated with this should be budgeted for. Facilities that are the first of their kind working towards GLP in their country or region may also need to invest significant time and effort into the development of facility-specific SOP training programmes. As more laboratories achieve GLP certification, there will be increased opportunities for inter-laboratory learning and training. Laboratories that are beginning their journey towards GLP compliance may, therefore, benefit from contacting those who have already completed the GLP certification process for advice on the project and for direction on where to access appropriate training and/or consultants.

Finally, funding bodies investing in capacity strengthening of laboratories in LMICs must consider the potentially increased cost and time scales necessary to achieve certification in a LMIC context. In order to maximise sustainability of such certifications, further investment is recommended in the supporting infrastructure at a national and regional level, including calibration laboratories and regional QMS expertise.

As the first insecticide test facility in Africa to achieve GLP certification, a case study of the processes to certification provides crucial information about how this was achieved, including all of the challenges encountered along the way. This knowledge can be applied to help accelerate progress towards GLP certification across the other six IVCC-supported test facilities in Africa, as well as other test facilities moving toward GLP certification in Asia and Central and South America that are being supported by WHO.

## Conclusion

The development, testing and registration of new insecticidal vector control products is a vital part of the global public health response to malaria, particularly in light of increasing resistance to insecticides in disease endemic countries. Non-clinical test facilities in these countries have a key role to play in this process, particularly in conducting laboratory and experimental studies to generate the data required by companies for product registration purposes. Therefore, ensuring that these test facilities can achieve GLP certification, the standard that will be required for studies to be accepted by WHO PQ-VCT, is a clear priority for the global malaria response. The standards required under GLP are rigorous, with an expected timeline of 24 months to completion. This study has shown that in LMICs, significantly more time may be required for the infrastructure improvements, recruitment and training of staff in key roles including Data Manager and QA Manager, SOP development and implementation, and integration with services external to the test facility including waste management, calibration and animal welfare. These challenges were successfully overcome by the GLP Project Management team at the test facility, and the recommendations to other test facilities on planning to minimise their affect are presented here. As the test facility is a non-government test facility, future research should consider what factors affect progress in both government and non-government test facilities, as well as similarities and differences in facilities in other African countries.

## Data availability

### Underlying data

Transcriptions of interviews with facility staff are available from the research group on request (please email ccr@lstmed.ac.uk to request access), on a case by case basis for the purpose of informing further research and on the condition that it will not be published in part or in entirety. They have not been made available as a dataset because they cannot be de-identified without compromising anonymity and the ethical approval conditions for the project stated that only the research team would have access to the data.

Harvard Dataverse: Developing laboratory capacity for Good Laboratory Practice certification: lessons from a Tanzanian insecticide testing facility - activities undertaken, https://doi.org/10.7910/DVN/AVCCBX ^31^.

This project contains the following underlying data:

- Activities_by_SANAS_Headings
- Activities_undertaken_with_timings

### Extended data

Harvard Dataverse: Developing laboratory capacity for Good Laboratory Practice certification: Lessons from a Tanzanian insecticide testing facility - extended data, https://doi.org/10.7910/DVN/MIDO06 ^32^.

This project contains the following extended data:

- Data underlying Figure 1 and Figure 3

Data are available under the terms of the Creative Commons Zero "No rights reserved" data waiver (CC0 1.0 Public domain dedication).

## References

[1] Lengeler C Sharp B: Indoor residual spraying and insecticide-treated nets. Reducing malaria’s burden: evidence of effectiveness for decision makers. Washington (D.C.): Global Health Council.2003;17–24.

[2] WHO: World malaria report 2019. World Health Organization: Geneva.2019 Reference Source

[3] Ranson H Lissenden N: Insecticide Resistance in African *Anopheles* Mosquitoes: A Worsening Situation That Needs Urgent Action to Maintain Malaria Control. *Trends Parasitol.* 2016;32(3):187–196. 10.1016/j.pt.2015.11.010 26826784

[4] Corbel V Durot C Achee NL: Second WIN International Conference on "Integrated Approaches and Innovative Tools for Combating Insecticide Resistance in Vectors of Arboviruses", October 2018, Singapore. *Parasit Vectors.* 2019;12(1):331. 10.1186/s13071-019-3591-8 31269996 PMC6610869

[5] Organisation for Economic Co-operation and Development: OECD Principles of Good Laboratory Practice (GLP) and GLP Compliance Monitoring.2019 Reference Source

[6] World Health Organization on behalf of the Special Programme for Research and Training in Tropical Diseases: Handbook: good laboratory practice (GLP): quality practices for regulated non-clinical research and development.2009 Reference Source

[7] World Health Organization on behalf of the Special Programme for Research and Training in Tropical Diseases: Good Laboratory Practice (GLP) Training Manual for the Trainer.2008 Reference Source

[8] World Health Organization on behalf of the Special Programme for Research and Training in Tropical Diseases: Good Laboratory Practice (GLP) Training Manual for the Trainee.2008 Reference Source

[9] Nkengasong JN Mbopi-Keou FX Peeling RW: Laboratory Medicine in Africa Since 2008: Then, Now, and the Future. *Lancet Infect Dis.* 2018;18(11):e362–e367. 10.1016/S1473-3099(18)30120-8 29980383 PMC13081755

[10] Gershy-Damet GM Rotz P Cross D: The World Health Organization African Region Laboratory Accreditation Process: Improving the Quality of Laboratory Systems in the African Region. *Am J Clin Pathol.* 2010;134(3):393–400. 10.1309/AJCPTUUC2V1WJQBM 20716795

[11] WHO Regional Office for Africa: WHO Guide for the Stepwise Laboratory Improvement Process Towards Accreditation in the African Region (SLIPTA).2015 Reference Source

[12] Datema TAM: Critical review of the Stepwise Laboratory Improvement Process Towards Accreditation (SLIPTA): suggestions for harmonization, implementation and improvement. *Trop Med Int Health.* 2012;17(3):361–367. 10.1111/j.1365-3156.2011.02917.x 22093245

[13] Royal Tropical Institute: Laboratory Quality Stepwise Implementation tool.2015 Reference Source

[14] Innovative Vector Control Consortium: Home | IVCC | Innovative Vector Control Consortium.2020 Reference Source

[15] South African National Accreditation System: CHECKLIST for GLP OECD No. 1.2009 Reference Source

[16] Palinkas LA Horwitz SM Green CA: Purposeful Sampling for Qualitative Data Collection and Analysis in Mixed Method Implementation Research. *Adm Policy Ment Health.* 2015;42(5):533–44. 10.1007/s10488-013-0528-y 24193818 PMC4012002

[17] Njelesani J Dacombe R Palmer T: A Systematic Approach to Capacity Strengthening of Laboratory Systems for Control of Neglected Tropical Diseases in Ghana, Kenya, Malawi and Sri Lanka. *Plos Negl Trop Dis.* 2014;8(3);e2736. 10.1371/journal.pntd.0002736 24603407 PMC3945753

[18] Gale NK Heath G Cameron E: Using the framework method for the analysis of qualitative data in multi-disciplinary health research. *BMC Medical Research Methodology.* 2013;13(1):117. 10.1186/1471-2288-13-117 24047204 PMC3848812

[19] Environment Directorate: OECD Principles of Good Laboratory Practice, in OECD Series on Principles of Good Laboratory Practice and Compliance Monitoring. *Organisation for Economic Co-operation and Development: Paris.* 1998 Reference Source

[20] Environment Directorate: GLP Consensus Document: The Application of GLP Principles to Computerised Systems, in OECD Series on Principles of Good Laboratory Practice and Compliance Monitoring. *Organisation for Economic Co-operation and Development: Paris.* 1995 Reference Source

[21] Environment Directorate: Advisory Document of the Working Group on Good Laboratory Practice, Application of GLP Principles to Computerised Systems, in OECD Series on Principles of Good Laboratory Practice and Compliance Monitoring. Organisation for Economic Co-operation and Development: Paris.2016 Reference Source

[22] WHO: Joint WHO – CDC Conference on Health Laboratory Quality Systems.2008 Reference Source 18689005

[23] Justman JE Koblavi-Deme S Tanuri A: Developing Laboratory Systems and Infrastructure for HIV Scale-Up: A Tool for Health Systems Strengthening in Resource-Limited Settings. *J Acquir Immune Defic Syndr.* 2009;52(Suppl 1):S30–S33. 10.1097/QAI.0b013e3181bbc9f5 19858935

[24] Zeh CE Inzaule SC Magero VO: Field Experience in Implementing ISO 15189 in Kisumu, Kenya. *Am J Clin Pathol.* 2010;134(3):410–418. 10.1309/AJCPZIRKDUS5LK2D 20716797

[25] Albert H Iragena JD Kao K: Implementation of quality management systems and progress towards accreditation of National Tuberculosis Reference Laboratories in Africa. *Afr J Lab Med.* 2017;6(2):490. 10.4102/ajlm.v6i2.490 28879161 PMC5523922

[26] Olmsted SS Moore M Meili RC: Strengthening Laboratory Systems in Resource-Limited Settings. *Am J Clin Pathol.* 2010;134(3):374–380. 10.1309/AJCPDQOSB7QR5GLR 20716792

[27] Miriam Schneidman RJD Carter J: Laboratory professionals in Africa: The Backbone of Quality Diagnostics.2014 Reference Source

[28] Kouriba B Oukem-Boyer OOM Traoré B: Installing biosafety level 3 containment laboratories in low- and middle-income countries: challenges and prospects from Mali's experience. *New Microbes New Infect.* 2018;26:S74–S77. 10.1016/j.nmni.2018.05.011 30402246 PMC6205564

[29] Sayed S Cherniak W Lawler M: Improving pathology and laboratory medicine in low-income and middle-income countries: roadmap to solutions. *Lancet.* 2018;391(10133):1939–1952. 10.1016/S0140-6736(18)30459-8 29550027

[30] Barbé B Verdonck K Mukendi D: The Art of Writing and Implementing Standard Operating Procedures (SOPs) for Laboratories in Low-Resource Settings: Review of Guidelines and Best Practices. *PLoS Negl Trop Dis.* 2016;10(11):e0005053. 10.1371/journal.pntd.0005053 27812100 PMC5094690

[31] Begg S Wright A Small G: Developing laboratory capacity for Good Laboratory Practice certification: lessons from a Tanzanian insecticide testing facility - activities undertaken.2020 10.7910/DVN/AVCCBX PMC7399503 32789289

[32] Begg S: Developing laboratory capacity for Good Laboratory Practice certification: Lessons from a Tanzanian insecticide testing facility - extended data.2020 10.7910/DVN/MIDO06 PMC7399503 32789289

