# Supplementary material for: Developing laboratory capacity for Good Laboratory Practice certification: lessons from a Tanzanian insecticide testing facility
Source: Gates Open Res. 2020 Jun 12;4:59. [Version 1] doi: 10.12688/gatesopenres.13133.1 (PMC7399503; doi:10.12688/gatesopenres.13133.1)
Supplement: Peer review history

# Peer review: Reviewer response for version 1

**DOI:** 10.21956/gatesopenres.14320.r28914

**Published:** 2020-08-03

**Authors:** Emmanuel Chanda

**Affiliations:** 1 Communicable and Non-Communicable Diseases Cluster, Tropical and Vector-borne Diseases, World Health Organization (WHO), Brazzaville, Democratic Republic of the Congo

Modern-day vector control for disease prevention is characterized by enduring constraints including diminishing effectiveness and sustainability due to various reasons. Given the prevailing challenges and the need for innovative and effective vector control tools, the WHO Prequalification Team for vector control products (WHO PQT-VCT) emphasizes availability of data for effective product evaluation from Good Laboratory Practice (GLP) certified test facilities. Begg and colleagues endeavor to assesses auditor GLP certification checklists and timings outlined in the WHO GLP Handbook in a Tanzanian insecticide-testing facility. The authors are applauded for expending efforts to meticulously stress challenges leading to the observed 23-month delay, and for highlighting the limitations of the study as well as providing the key lessons learnt to inform achievement of the rigorous standards for GLP certification in compliance with the WHO PQ-VCT requirements.

 It is commendable that the first insecticide-testing facility in Africa has achieved GLP certification, albeit beyond the expected timelines and amidst daunting challenges experienced in low- and middle-income countries (LMICs). The study is timely and stresses critical aspects that would merit the updating of the WHO GLP Handbook. Particularly; the underemphasized computer systems in auditor GLP checklists despite their broad applicability; the underestimated 24-month timeline requirement in the stepwise approach to achieving GLP certification for key areas that require substantial time and effort investments to be achieved; and, the non-inclusion of construction and infrastructure changes in the GLP stepwise approach. Apart from emphasizing that construction and infrastructure changes need to be included in the WHO GLP Handbook stepwise approach, the authors have not clearly recommended revision of the WHO GLP Handbook. While lack of clarity on context-specific findings and those to be generalized across governments and non-government sites and contexts is well noted and precludes generality of findings, there is great need for robust gap analyses, extension of the required timelines and inclusion of additional areas in the WHO GLP Handbook. If GLP certification is to be optimized for other LMICs, the findings should inform the updating of the WHO GLP Handbook as an overall guidance document.

 I find the article to be well written and provides an example epitome for subsequent in-depth assessments of other test facilities in Africa and other regions to facilitate timely attainment of GLP certification and bringing to market of new vector control tools.

Is the work clearly and accurately presented and does it cite the current literature?

Yes

If applicable, is the statistical analysis and its interpretation appropriate?

Yes

Are all the source data underlying the results available to ensure full reproducibility?

Yes

Is the study design appropriate and is the work technically sound?

Yes

Are the conclusions drawn adequately supported by the results?

Yes

Are sufficient details of methods and analysis provided to allow replication by others?

Yes

Reviewer Expertise:

Medical Entomology and Vector Control

I confirm that I have read this submission and believe that I have an appropriate level of expertise to confirm that it is of an acceptable scientific standard.

# Peer review: Reviewer response for version 1

**DOI:** 10.21956/gatesopenres.14320.r28916

**Published:** 2020-06-29

**Authors:** John Gimnig

**Affiliations:** 1 Centers for Disease Control and Prevention, Atlanta, GA, USA

This manuscript describes the challenges in moving a single laboratory in Africa to Good Laboratory Practice (GLP) certification. While the WHO GLP handbook indicates that certification can be implemented in 24 months, the certification process in this laboratory took 47 months, suggesting the proposed timeline may be unrealistic for low and middle income countries. Through document review and semi-structured interviews with staff in the laboratory, including laboratory technicians and administrative staff as well as middle and senior level scientific research staff, the authors identified challenges and bottlenecks to GLP certification. Of the 45 steps identified in the WHO GLP handbook, 27 (60%) took longer than outlined with five taking more than 1 year longer than indicated. Major delays included identification of appropriate trainers in GLP, the hiring of qualified staff to implement GLP, the need for development of multiple SOPs from scratch, upgrades to facilities, the need to acquire GLP compliant items from outside the country and the lack of national guidelines needed for GLP compliance. 

 The manuscript was well written and provides a valuable resource for other laboratories in LMIC countries that plan to become GLP certified. The experimental design (document review and semi-structured interviews), is adequate for the purpose of this study. The results are presented clearly and accurately and the conclusions are justified and supported by the data.

 The only minor criticism I have of the manuscript was that some of the descriptions of tasks and their timelines were not clear. For example, the authors mention that steps 7.1 to 7.9 of the WHO GLP handbook should start 18 months after beginning the certification process and take 3 months. However, it took 38 months to complete this in the laboratory described in this paper. If this was started at 18 months and took 38 months to complete, that would have been completed 9 months after the certification process was reported completed (56 months versus 47 months). It might be helpful to outline each step with a targeted starting point, actual start, targeted end point and actual end point for each step.

Is the work clearly and accurately presented and does it cite the current literature?

Yes

If applicable, is the statistical analysis and its interpretation appropriate?

Not applicable

Are all the source data underlying the results available to ensure full reproducibility?

Partly

Is the study design appropriate and is the work technically sound?

Yes

Are the conclusions drawn adequately supported by the results?

Yes

Are sufficient details of methods and analysis provided to allow replication by others?

Yes

Reviewer Expertise:

Medical entomology and vector biology

I confirm that I have read this submission and believe that I have an appropriate level of expertise to confirm that it is of an acceptable scientific standard.
